# A Quantile Regression Approach to Estimating the Distribution of Anesthetic Procedure Time during Induction

**DOI:** 10.1371/journal.pone.0134838

**Published:** 2015-08-04

**Authors:** Hsin-Lun Wu, Wen-Kuei Chang, Ken-Hua Hu, Richard M. Langford, Mei-Yung Tsou, Kuang-Yi Chang

**Affiliations:** 1 Department of Anesthesiology, Taipei Veterans General Hospital and School of Medicine, National Yang-Ming University, Taipei, Taiwan; 2 Department of Anesthesiology, Hsinchu Mackay Memorial Hospital, Hsinchu, Taiwan; 3 Pain and Anaesthesia Research Centre, William Harvey Research Institute, Barts and The London School of Medicine and Dentistry, Queen Mary College, University of London, London, EC1A 7BE, United Kingdom; University of Colorado Denver, UNITED STATES

## Abstract

Although procedure time analyses are important for operating room management, it is not easy to extract useful information from clinical procedure time data. A novel approach was proposed to analyze procedure time during anesthetic induction. A two-step regression analysis was performed to explore influential factors of anesthetic induction time (AIT). Linear regression with stepwise model selection was used to select significant correlates of AIT and then quantile regression was employed to illustrate the dynamic relationships between AIT and selected variables at distinct quantiles. A total of 1,060 patients were analyzed. The first and second-year residents (R1-R2) required longer AIT than the third and fourth-year residents and attending anesthesiologists (*p* = 0.006). Factors prolonging AIT included American Society of Anesthesiologist physical status ≧ III, arterial, central venous and epidural catheterization, and use of bronchoscopy. Presence of surgeon before induction would decrease AIT (*p* < 0.001). Types of surgery also had significant influence on AIT. Quantile regression satisfactorily estimated extra time needed to complete induction for each influential factor at distinct quantiles. Our analysis on AIT demonstrated the benefit of quantile regression analysis to provide more comprehensive view of the relationships between procedure time and related factors. This novel two-step regression approach has potential applications to procedure time analysis in operating room management.

## Introduction

Monitoring procedure time is essential for operating room (OR) efficiency improvement and setting corresponding performance standards in time domain is beneficial to identify unusual events which may prolong procedure time and result in OR inefficiency.[1–3] With this information, the performance of individual specialists can be evaluated from the time perspective and extents and reasons of prolonged procedure time can be unveiled. Although procedure time data may be readily accessible from OR information systems, how to extract useful information from these data without an appropriate analytic approach is not so intuitive. First of all, these data are not collected for study purpose and subject to miscellaneous confounding effects. The second problem is that a procedure may be composed of different combinations of sub-procedures determined case by case. For example, an arterial catheterization may be necessary for some patients but not all during the induction of general anesthesia. As a rule, only the overall procedure time, instead of individual sub-procedure time, is available. Thus it is difficulty to evaluate the influence of each sub-procedure on the total procedure time. Third, the distribution of procedure time is not easy to identify and the upper tail of procedure time distribution provides more valuable information about causes of the prolongation of procedure time than the mean does. However, most of parametric statistical models which can be used to analyze procedure time typically focus on conditional mean but ignore both tails of a response distribution. This is clearly unfavorable to the analysis of procedure time data since the upper tail of the procedure time distribution is of primary interest. Besides, common analytic approaches often impose strict assumptions on how the covariates are permitted to affect event time, like the covariates can only affect the location but not the shape of event time distribution, and fail to characterize the dynamic relationships between outcome and predictor variables.[4]

In order to solve the analytic problems of procedure time data, a novel two-step approach was proposed to the analysis of procedure time data using a quantile regression approach.[5] The quantile regression analysis allows us to focus on the evaluation of covariate effects at specific quantiles of conditional procedure time distribution without the risk of biased results due to the robustness of quantile regression to distributional assumptions.[4, 5] In this study, anesthetic induction time (AIT) collected from clinical practice was analyzed as an example to demonstrate benefits of this two-step regression approach to procedure time analyses. At first, we used the linear regression analysis to find out correlates of AIT and estimate their mean effects on AIT. Afterward we employed the quantile regression analysis to evaluate influences of selected variables on AIT at distinct quantiles. Predicted values of AIT under miscellaneous conditions at distinct quantiles of AIT distribution were obtained and compared with predicted AIT from linear regression analyses. These predicted values can be used to establish performance standards in time domain for combinations of various anesthetic procedures under diverse conditions.

## Materials and Methods

### Setting of the Study

We investigated AIT by chart review in 25 ORs of Taipei Veterans General Hospital with the approval of our Institutional Review Board (VGHIRB No.: 2011-11-009-IC). Written informed consent was waived and patient information was anonymized and de-identified prior to analysis. The surgical services studied include thoracic, general, urological, colorectal, plastic, orthopedic, and neurological surgeries. Only inpatient surgeries were included in the survey. Cases performed by surgeons under local anesthesia were excluded. Patients who came with endotracheal tube in place were also eliminated from the analysis.

### Potential Factors Related to AIT for Data Collection

The AIT is defined as the presence of anesthesiologist in OR with facility and patient ready to begin anesthesia until the accomplishment of anesthetic procedures which have put patients to a certain degree of anesthetic level and may proceed to surgical positioning.[6] The waiting time for the anesthesiologists was not considered in this study. AIT was recorded in minutes by nurse anesthetists in each OR. Factors which might be associated with AIT were recorded for each patient. The anesthetic methods include general and regional anesthesia. The use of laryngeal mask airway and bronchoscope for fiberoptic-assisted tracheal intubation or examination of double-lumen endobronchial tube were further isolated from GA group for analysis. Both spinal anesthesia and axillary block were categorized into the RA group. Other data collected include American Society of Anesthesiologist (ASA) physical status and application of invasive procedures such as arterial, central venous and epidural catheterization. ASA physical status was dichotomized into two groups (ASA I-II vs. ASA III-IV). In addition, the presence of surgeon before anesthetic induction was also recorded as a potentially influential factor of AIT (S1 Dataset).

To evaluate level of training on performance, the main conductor of anesthesia was classified into 3 subgroups based on their clinical experiences: attending anesthesiologist with qualified certificate, year-3 to -4 residents (R3-R4), and year-1 to -2 residents (R1-R2). During the study period, there were 16 attending anesthesiologists and 17 residents. If the case was conducted by a resident initially, the case would remain in the original resident group despite aids from other senior residents or attending anesthesiologists.

### Data Analysis

AIT was log-transformed and presented as geometric mean with 95% confidence interval (CI) due to the right-skewed distributional property. Categorical data were described as count with percentage (%). One-way analysis of variance was used to compare the mean values of log-transformed AIT among different training levels of anesthesiologists. Anesthetic procedures performed by anesthesiologists with different training levels were compared using Chi square test. Linear regression analysis was conducted to select significant predictors of log-transformed AIT using a forward stepwise model selection strategy with the entry criterion of 0.05 and removal criterion of 0.1 in significance level. The coefficient of determination (*R* ^2^) and adjusted *R* ^2^ were also calculated for the selected model. Duan's smearing estimate was used to retransform AIT back to its original scale to compensate for the potential bias from retransformation.[7] The candidate variables included regional anesthesia, ASA physical status ≧ III, training level of anesthesiologists, invasive procedures (arterial, central venous or epidural catheterization, or bronchoscopy), surgical types (colorectal, thoracic, general, plastic, orthopedic, neurologic, and genitourinary surgeries), and surgeons’ presence before anesthetic induction (0 = No and 1 = Yes). Furthermore, the ordered AIT distribution was divided into 20 equal-sized data subsets and the quantiles reflected the boundary values between consecutive subsets and quantile regression models with bootstrapped standard errors were used to estimate the effects of selected factors at selected quantiles of AIT, including 0.1, 0.25, 0.5, 0.75 and 0.9. Goodness of fit for the quantile regression model was assessed with pseudo-*R* ^2^. With respect to the sample size requirement of stepwise model selection for the linear regression, Tabachnick and Fidell suggested that the minimum number of cases should be more than 40 × *m*, where *m* is the number of candidate variables in the model.[8, 9] Given the case number of 1060, this criterion was met in our analysis. A *p* value less than 0.05 was considered to be statistically significant. All statistical analyses were performed with Stata 12.0 (Stata Corp, College Station, USA).

## Results

There were 1,060 patients included in this analysis. Table 1 compares AIT, ASA physical status of patients, and anesthetic procedures performed by different groups of anesthesiologists. Four hundred anesthetic procedures were implemented by the attending anesthesiologists. The R1-R2 residents performed another 297 cases and the R3-R4 residents completed the remaining 363 anesthesia. The geometric means of AIT for the three groups of R1-R2 and R3-R4 residents and attending anesthesiologists were 15.5, 14.1 and 14.9 minutes, respectively. Note that there was no significant difference in AIT, the percentage of patients with ASA physical status ≧ III, anesthetic method (general or regional anesthesia), and invasive procedures (i.e. arterial, central venous and epidural catheterization, and bronchoscopy) among these three groups of anesthesiologists.

**Table 1:** Comparisons of anesthetic induction time, type of anesthesia and related procedures among different groups.

	Attending anesthesiologist		R1-R2		R3-R4			Total	
	(*n* = 400)		(*n* = 297)		(*n* = 363)			(*n* = 1060)	
	Count	%	Count	%	Count	%	*p*	Count	%
	(GM)	(95% CI)	(GM)	(95% CI)	(GM)	(95% CI)		(GM)	(95% CI)
Anesthetic induction time	(14.9)	(14–15.9)	(15.5)	(14.3–16.9)	(14.1)	(13.2–15.1)	0.21	(14.1)	(10–25)
General anesthesia	305	76.3%	211	71.0%	277	76.3%		793	74.8%
Laryngeal mask airway	13	3.3%	21	7.1%	17	4.7%	0.07	51	4.8%
Bronchoscopy	31	7.8%	30	10.1%	22	6.1%	0.16	83	7.8%
Regional anesthesia	95	23.8%	86	29.0%	86	23.7%	0.21	267	25.2%
ASA ≧ III	123	30.8%	82	27.6%	116	32.0%	0.47	321	30.3%
Arterial catheterization	191	47.8%	141	47.5%	153	42.1%	0.23	485	45.8%
Central venous catheterization	146	36.5%	95	32.0%	111	30.6%	0.19	352	33.2%
Combined epidural anesthesia	37	9.3%	13	4.4%	27	7.4%	0.05	77	7.3%

R1-R2 = year-1 to -2 resident; R3-R4 = year-3 to -4 resident; GM = geometric mean; CI: confidence interval; Bronchoscopy was used for fiberoptic-assisted tracheal intubation or examination of double lumen endobronchial tube; ASA = American Society of Anesthesiologist physical status

Table 2 illustrates the effects of selected factors on log-transformed AIT evaluated with the linear regression analysis. For a patient receiving an orthopedic surgery with ASA physical status I or II, the mean time for an attending anesthesiologist to complete induction of general or regional anesthesia without additional anesthetic procedures was around 12.1 minutes (the baseline condition). ASA physical status ≧ III and additional anesthetic procedures including arterial, central venous and epidural catheterization, and bronchoscopy, would significantly increase AIT. For example, an additional arterial catheterization would result in 47% increase in AIT from the baseline condition and extra arterial and central venous catheterizations would double the AIT for the baseline condition (12.1 × *e* ^(0.38 + 0.33)^). In contrast, presence of surgeon before induction significantly reduced AIT by 27% (*p* < 0.001). Types of surgery also had significant influence on AIT (*p* < 0.001). On average, genitourinary and colorectal surgery required significantly less induction time than orthopedic surgery (both *p* < 0.001). There was no significant difference in AIT between other surgical types and orthopedic surgery. Different training level of anesthesiologists also had significant influence on AIT (*p* < 0.001). The R1-R2 residents required more time for induction than attending anesthesiologists (12% increase in AIT on average, *p* = 0.003). In contrast, no significant difference in AIT was noted between R3-R4 residents and attending anesthesiologists (*p* = 0.82). Regional anesthesia was not selected as an influential factor of AIT in the linear regression analysis (*p* = 0.31). The *R* ^2^ and adjusted *R* ^2^ of the final selected model were 0.42 and 0.41, respectively.

**Table 2:** Selected factors associated with anesthetic induction time by linear regression analysis.

Factor	*β*	SE	*p*	exp(*β*)	95% CI	
Training level			0.002			
R1-R2	0.12	0.04	0.003	1.12	1.04	~1.21
R3-R4	-0.01	0.04	0.819	0.99	0.92	~1.07
Attending anesthesiologist (reference group)						
Surgical types			<0.001			
Colorectal surgery	-0.30	0.06	<0.001	0.74	0.66	~0.84
General surgery	0.08	0.05	0.084	1.08	0.73	~1.03
Genitourinary surgery	-0.19	0.05	<0.001	0.82	0.99	~1.18
Neurosurgery	0.09	0.05	0.072	1.10	0.74	~0.91
Plastic surgery	0.09	0.07	0.207	1.09	0.99	~1.22
Thoracic surgery	-0.14	0.09	0.111	0.87	0.95	~1.26
Orthopedic surgery (reference group)						
ASA ≧ III vs. ASA I-II	0.09	0.03	0.009	1.09	1.02	~1.17
Arterial catheterization	0.38	0.05	<0.001	1.47	1.34	~1.61
Central venous catheterization	0.33	0.05	<0.001	1.39	1.27	~1.53
Combined epidural anesthesia	0.37	0.06	<0.001	1.45	1.28	~1.64
Bronchoscopy	0.29	0.09	0.001	1.33	1.13	~1.58
Presence of surgeon before induction	-0.32	0.06	<0.001	0.73	0.65	~0.81
*Constant*	2.36	0.04	<0.001	10.64	9.84	~11.52

*β* = regression coefficient; SE = standard error of regression coefficient; Factors with *β* < 0 shortened anesthetic induction time and those with *β* > 0 prolonged anesthetic induction time. The *R* ^2^ and adjusted *R* ^2^ are 0.42 and 0.41, respectively. R1-R2 = year-1 to -2 resident; R3-R4 = year-3 to -4 resident; ASA = American Society of Anesthesiologist physical status; Bronchoscopy was used for fiberoptic-assisted tracheal intubation or examination of double lumen endobronchial tube.

Quantile regression analyses at 0.1, 0.25, 0.5, 0.75, and 0.9 quantiles of AIT distribution is shown in table 3. For distinct training levels of anesthesiologists, R1-R2 demanded 4 and 6 more minutes of AIT than attending anesthesiologists did at the quantiles of 0.75 and 0.9, respectively. There was no significant difference in AIT between R3-R4 and attending anesthesiologists at all selected quantiles. AIT for patients with ASA ≧ III was 3 and 7 minutes longer than for patients with ASA I-II at the quantiles of 0.75 and 0.9. There was no significant difference in AIT between different ASA physical statuses at lower quantiles. All anesthetic procedures including arterial, central venous and epidural catheterization, and bronchoscopy would significantly increase AIT throughout all selected quantiles. Arterial catheterization demanded 3 minutes at the quantile of 0.1, 4 minutes at the quantile of 0.25, and about 6 minutes thereafter. Central venous catheterization required 5 minutes at the quantile of 0.1, 6 minutes at the quantile of 0.25, and near 7 minutes at the quantile of 0.5, 8 minutes at the quantile of 0.75 and 9 minutes at the quantile of 0.9. The use of bronchoscope consumed additional 9 to 16 minutes at the quantiles of 0.75 to 0.9. Presence of surgeon before anesthetic induction could reduce AIT from 2 to 6 minutes. However, this effect was not significant at the quantiles of 0.1 and 0.75. Surgical types also had significant influence on AIT. Induction time for colorectal surgery was significantly less than orthopedic surgery (from 3 to 7, all *p* < 0.05). There was no significant difference in induction time between plastic and orthopedic surgeries. Neurologic and general surgeries demanded 2 more minutes of AIT than orthopedic surgery at the quantiles of 0.1 and 0.25. Genitourinary surgery demanded about 3 minutes less AIT than reference group at the quantiles of 0.25 and 0.5. Thoracic surgery required 3 minutes less AIT at the quantile of 0.5, 5 minutes less at the quantile of 0.75, and 12 minutes less at the quantile of 0.9.

**Table 3:** Estimates of factors associated with anesthetic induction time by quantile regression at 0.1, 0.25, 0.5, 0.75 and 0.9 quantiles.

Quantile	0.1			0.25			0.5			0.75			0.9		
	*β*	SE	*p*	*β*	SE	*p*	*β*	SE	*p*	*β*	SE	*p*	*β*	SE	*p*
Training level															
R1-R2	0	0.59	1	0	0.47	1	1.44	0.84	0.085	4	1.46	0.006	6	2.04	0.003
R3-R4	0	0.37	1	0	0.41	1	-0.56	0.62	0.372	0	1.01	1	1	1.72	0.561
Attending anesthesiologist (reference group)															
Surgical types															
Colorectal surgery	-3	1.45	0.039	-3	0.92	0.001	-4.22	0.89	< 0.001	-7	1.44	< 0.001	-7	2.23	0.002
General surgery	0	1.95	1	-2	1.63	0.219	-3.22	1.59	0.043	-5	2.54	0.049	-12	3.73	0.001
Genitourinary surgery	2	1.01	0.047	2	0.91	0.027	0.44	0.75	0.555	0	1.16	1	0	2.21	1
Neurosurgery	0	0.63	1	-3	0.78	< 0.001	-2.78	1.12	0.013	-1	1.93	0.604	-1	2.88	0.728
Plastic surgery	2	0.98	0.043	2	0.97	0.038	1.44	1.18	0.22	1	1.71	0.559	2	2.72	0.462
Thoracic surgery	1	1.14	0.381	1	0.93	0.284	0	1.1	1	0	2.35	1	0	2.96	1
Orthopedic surgery (reference group)															
ASA ≧ III vs. ASA I-II	0	0.36	1	0	0.48	1	1.22	0.7	0.079	3	1.48	0.044	7	2.11	0.001
Arterial catheterization	3	1	0.003	4	0.93	< 0.001	6.22	1.12	< 0.001	6	1.57	< 0.001	6	2.2	0.006
Central venous catheterization	5	0.97	< 0.001	6	1.07	< 0.001	6.78	1.14	< 0.001	8	1.64	< 0.001	9	2.38	< 0.001
Combined epidural anesthesia	5	0.71	< 0.001	4	1.28	0.002	5.33	1.08	< 0.001	6	1.86	0.001	7	2.91	0.016
Bronchoscopy	5	1.74	0.004	5	1.4	< 0.001	4.33	1.54	0.005	9	2.63	0.001	16	5.95	0.007
Presence of surgeon before induction	-2	1.09	0.066	-3	0.9	0.001	-2.22	1	0.026	-3	1.76	0.088	-6	1.72	< 0.001
*constant*	5	0.42	< 0.001	8	0.8	< 0.001	10.56	0.56	< 0.001	15	0.91	< 0.001	20	1.76	< 0.001
Pseudo-*R* ^2^	0.19			0.23			0.26			0.24			0.23		

*β* = regression coefficient; SE = bootstrapped standard error of regression coefficient; pseudo-*R* ^2^ = fit statistics of quantile regression at specific quantiles. R1-R2 = year-1 to -2 resident; R3-R4 = year-3 to -4 resident; ASA = American Society of Anesthesiologist physical status; Bronchoscopy was used for fiberoptic-assisted tracheal intubation or examination of double lumen endobronchial tube.

Table 4 presents some examples of estimated AIT from the linear and quantile regression analyses under miscellaneous conditions. The linear regression analysis provided only one conditional mean estimate of AIT under a specific condition. In contrast, the quantile regression analysis could generate different estimates of AIT at distinct quantiles under the same condition. Note that the mean AIT estimates from the linear regression analysis are similar to their median counterparts from the quantile regression analysis for simpler anesthetic cases but the estimated mean AIT was remarkably greater than the corresponding median estimates. In addition, the obtained AIT estimates at a specific quantile under miscellaneous conditions could be used as a reference table. For example, the induction of a simple general or regional anesthesia without other invasive procedures for a patient with ASA < III should be completed in 19 and 15 minutes by R1-R2 and R3-R4 residents in 75% of cases, respectively. On average, induction of cases with ASA physical status ≧ III would need an extra of 1, 3 and 7 minutes at the quantiles of 0.5, 0.75 and 0.9, respectively. For more complicated cases with various combinations of anesthetic procedures, AIT can also be estimated accordingly using the quantile regression analysis in a similar manner.

**Table 4:** A reference table of anesthetic induction time needed for various combinations of procedures for residents with different training levels at the 0.5, 0.75 and 0.9 quantiles based on the linear and quantile regression analyses.

Training level	R1-R2								R3-R4							
ASA physical status	I-II				≧ III				I-II				≧ III			
Standard (quantile)	Mean	0.5	0.75	0.9	Mean	0.5	0.75	0.9	Mean	0.5	0.75	0.9	Mean	0.5	0.75	0.9
Simple general or regional anesthesia	**14**	12	19	26	**15**	13	22	33	**12**	10	15	21	**13**	11	18	28
+ arterial catheterization	**20**	18	25	32	**22**	19	28	39	**18**	16	21	27	**19**	17	24	34
+ central venous catheterization	**19**	19	27	35	**21**	20	30	42	**17**	17	23	30	**18**	18	26	37
+ epidural catheterization	**20**	17	25	33	**21**	19	28	40	**17**	15	21	28	**19**	17	24	35
+ arterial and central venous catheterizations	**28**	25	33	41	**30**	26	36	48	**24**	23	29	36	**27**	24	32	43
+ arterial and epidural catheterizations	**29**	24	31	39	**32**	25	34	46	**25**	22	27	34	**28**	23	30	41
+ arterial, central venous and epidural catheterizations	**40**	30	39	48	**44**	32	42	55	**35**	28	35	43	**39**	30	38	50
+ arterial and central venous catheterizations + bronchoscopy	**37**	29	42	57	**40**	31	45	64	**33**	27	38	52	**36**	29	41	59

R1-R2 = year-1 to -2 resident; R3-R4 = year-3 to -4 resident; ASA = American Society of Anesthesiologist physical status; bronchoscopy = fiberoptic-assisted tracheal intubation or examination of double lumen endobronchial tube. The numbers in boldface are estimated AIT based on results of the linear regression analysis and retransformed into the original scale with Duan’s smearing factor. Only conditional mean estimates of AIT can be obtained using the linear regression analysis. In contrast, it is possible to estimate anesthetic induction time under miscellaneous conditions at distinct quantiles using the quantile regression analysis. For example, if a simple general or regional anesthesia without any invasive procedures for a patient with ASA < III was completed by an R1-R2 resident in 20 minutes, we know that he or she finished this task slower than at least 75% of his or her peers (19 minutes at the quantile of 0.75). Similarly, AIT for various combinations of anesthetic procedures at distinct quantiles can also be estimated using this analytical approach. Note that the predicted mean AIT from the linear regression analysis are greater their median counterparts from the quantile regression analysis. This implies the right-skewed property of AIT distribution and quantile regression analysis can provide more comprehensive information throughout the whole distribution of AIT under miscellaneous conditions.

## Discussion

This study applied a two-staged analytic approach to investigate factors affecting AIT in OR. This way of data exploration has several advantages over the traditional approaches. At first, a linear regression approach was used to screen the influential factors of AIT and estimate the mean effects of selected variables on AIT. From the economic and managerial perspectives, the conditional mean of AIT estimated from linear regression analyses can be applied to further inferences regarding OR efficiency or management.[10–12] Therefore, linear regression analyses used in the first stage have the potency of further applications to economic analyses. Unfortunately, heterogeneity and right-skewed property of AIT distribution confined the utility of pure linear regression analyses. Another problem of the linear regression analysis is that they focus on central tendency and mean effects of explanatory variables. However, for AIT analyses, it is the prolonged AIT, not the mean, that interests us after all. In contrast, a quantile regression approach allows for complete examination of influences of influential factors on the entire distribution of AIT and thus details about the prolonged AIT (the upper tail of the distribution) could be inspected more explicitly. It provides more comprehensive view and dynamic features of the relationships between AIT and its influential factors. The establishment of performance standards in time domain for procedures in OR based on the results of quantile regression analyses also becomes feasible. Our study demonstrated the versatility of quantile regression to investigate factors related to AIT, evaluate their effects on AIT, and predict AIT under various conditions by a thorough analysis.

According to the results of quantile regression analysis, training level only exerted significant effects at quantiles above the median of AIT. R1-R2 required more minutes than senior anesthesiologists for more complicated cases. Devoid of experiences and proficiency in anesthetic management may account for this discrepancy. In contrast, R3-R4 could perform anesthetic induction as fast as attending physicians, even for more complicated cases. Our findings agree with other studies which concluded that junior anesthetic trainees in OR may prolong AIT.[6, 10, 13] The merit of our findings is to provide more details regarding the relations between AIT and training level along different quantiles of AIT.

In our analysis, patients with ASA physical status ≧ III required significant longer time for anesthetic induction. A study about the pre-incision surgical period also demonstrated that anesthesia release time (from patient on the OR table to completion of anesthetic induction and beginning of surgical positioning) increased significantly across ASA physical status I-IV. On the other hand, few studies compared AIT for different surgeries.[14, 15] The reason may be complicated involving disease entity, surgical procedure, anesthetic technique, patient characteristics, and surgeon performance, etc. Nevertheless, this information still have potential implications for OR management. For example, patients with colorectal surgery can be transferred to OR later since less time would be needed to complete anesthetic induction for them.

Although it could be expected that combining extra anesthetic procedures would increase AIT substantially, estimating AIT of various combinations of anesthetic procedures at distinct quantiles is by no means easy using a traditional analytical approach. According to the results of quantile regression analysis, we found that cases the extra time required to complete anesthetic induction for cases with additional arterial catheterization remained constant from the median to the upper end of AIT distribution. For cases needing epidural or central venous catheterization, the time required to complete anesthetic induction increased gradually with the progression of quantiles. In contrast, the extra time required to complete anesthetic induction with bronchoscopy increased exponentially with the advance of quantiles from the median to the upper end of AIT distribution. These may imply that difficulty in arterial catheterization was a rare cause of prolonged AIT but difficulty in central venous or epidural catheterization did contribute to the prolongation of AIT. For cases which needed bronchoscopy, it have greater potential to dramatically lengthen AIT. These findings from the quantile regression analysis can provide useful information about residency training and OR management.

With the aid of quantile regression analysis on AIT, a time standard for procedures can be set up for various purposes. Cases with AIT exceeding pre-determined percentiles, such as the 75^th^ or 90^th^ percentiles, should be reported and investigated for reasons. For example, technical difficulty and unfamiliarity can be improved by further training programs such as simulator for junior anesthesiologists.[16, 17] Moreover, the efficacy of training programs could also be assessed by re-estimation of related parameters with quantile regression analysis after training. Our analytic approach provides a useful strategy to find out delaying factors of AIT in OR and evaluate efficacy of a training program.

Limitations in our study are discussed below. First, it is difficult to individually record the time required to complete each anesthetic procedure. Therefore, a model-based approach was used to estimate the time spent for each procedures. Discrepancy between actual and estimated time is possible. Second, the variables selected in our analysis only explained the variance of AIT in part. More explanatory variables like autonomy of residents and intervention of attending physician should be considered in future studies related to AIT.

## Conclusions

We proposed a two-stage analytic approach to explore factors affecting AIT in OR. Linear regression was used at first to screen influential factors and estimate mean effects on AIT, and then quantile regression was employed to further evaluate effects of selected factors on AIT at distinct quantiles and to establish performance standards in time domain under various conditions. Our study demonstrated the usefulness of quantile regression to disclose the complex relationships between AIT in OR and their influential factors. This novel approach has promising applications to procedure time analyses in OR and provides valuable information for OR management.

## Supporting Information

S1 Dataset Dataset for the analysis of anesthetic induction time. (XLS)
